# The natural history of interferon-α induced thyroiditis in chronic hepatitis c patients: a long term study

**DOI:** 10.1186/1756-6614-4-2

**Published:** 2011-01-08

**Authors:** Huy A Tran, Tracey L Jones, Elizabeth A Ianna, Glenn EM Reeves

**Affiliations:** 1 Hunter Area Pathology Service and University of Newcastle, Locked Bag Number 1, Hunter Mail Region Centre, Newcastle, New South Wales 2310, Australia; 2 Hepatitis C Service, Gastroenterology Department, John Hunter Hospital and University of Newcastle, Locked Bag Number 1, Hunter Mail Region Centre, Newcastle, New South Wales 2310, Australia; 3 Hepatitis C Service, Gastroenterology Department, John Hunter Hospital, Locked Bag Number 1, Hunter Mail Region Centre, Newcastle, New South Wales 2310, Australia; 4 Hunter Area Pathology Service and University of Newcastle, Locked Bag Number 1, Hunter Mail Region Centre, Newcastle, New South Wales 2310, Australia

## Abstract

**Background:**

Autoimmune thyroid disease is a common complication of patients with chronic hepatitis C undergoing combination pegylated interferon-α and ribavirin treatment. A small proportion develops interferon-induced thyroiditis of which the long term natural history is unknown and how it compares with de novo thyroiditis. The aim of the study is to determine the natural history of thyroid disease including antibody profile in this particular setting 36 months from the completion of therapy.

**Methods:**

A cohort of 18 hepatitis C patients (mean age 45 ± 8 years (standard deviation)) who developed exclusively thyroiditis in this setting was followed every 12 months after the completion of therapy for 36 months. Investigations included thyrotropin, free tetra-iodothyronine, free tri-iodothyronine levels and thyroid autoantibodies.

**Results:**

None of the patients developed any long term thyroid disease. Two patients had a prolonged hypothyroid phase of the thyroiditis early after the completion of treatment but recovered fully. The remaining 16 patients remained euthyroid. Similarly, thyroid autoantibodies all declined and returned to reference range.

**Conclusions:**

The long term natural history in this small series of interferon induced thyroiditis was benign. If a larger series confirms a similar outcome then there is no long term residual effect on thyroid function and follow-up testing would not be warranted.

## Background

Hepatitis C remains one of the major causes of chronic liver infection and cirrhosis worldwide. The most effective and established treatment available for this condition is the combination ribavirin and pegylated interferon-α (IFN-α). A major and common adverse effect of this treatment is the development of thyroid disease during therapy. A large spectrum of autoimmune thyroid disease (TD) has been described to occur ranging from Graves' disease to thyroiditis to frank primary hypothyroidism [1-3]. As the number of patients undertaking treatment is expected to rise, a proportion of these patients will progress to develop thyroid disease. It is therefore important to understand and fully clarify the natural progression of the disease. This forms a critical part of the long term management and counselling for these patients. It has been reported that ~50% of these patients recover from this complication [4,5]. Furthermore, other reports are retrospective and follow patients in an ad hoc fashion with very variable and unstandardised duration. In addition, some of the studied groups included the use of IFN as monotherapy and regular IFN (rather than pegylated) in combination with ribavirin [6]. In some cases, patients remain permanently thyroxine dependent although no withdrawal trials were attempted. The long term natural history of this condition beyond this time frame remains largely unknown and it is uncertain whether it is different from other forms of thyroiditides arising de novo.

The aim of this report is to follow prospectively the long term natural history of TD 36 months after the completion of treatment in a cohort of patients who developed thyroid disease during therapy with pegylated interferon and also to compare the outcome with the natural history of the thyroiditides arising de novo.

## Methods

### Patients

The patients are all recruited from a Hepatitis C service centre in a major tertiary referral hospital. A total of 18 patients were available for study. These were retrieved from a pool of 358 patients over a 5 year period, giving an annual incidence of ~5.0%. All were followed-up over a 36-month period. All were medication naive, i.e. all were undergoing therapy for the first time. All other causes of chronic hepatitis were excluded including hepatitis B and chronic alcoholic liver disease. Baseline characteristics of all studied subjects are summarised in Table 1.

**Table 1:** Baseline characteristics, hepatitic outcomes and thyrotropin outcome profiles in all 18 thyroiditis patients.

*Subject* *No*.	*Gender*	*Age*	*Hepatitis C Genotype*	*Duration of Therapy (weeks)*	*SVR*	*End of Rx TSH*	*TSH at 12 months*	*TSH at 24 months*	*TSH at 36 months*
1	M	26	1a	48	Y	2.1	1.8	2.8	3.4

2	M	51	2	24	Y	1.7	3.2	3.1	2.7

3	M	54	3	24	Y	3.8	2.3	2.2	3.2

4	M	49	4	48	Y	1.1	1.4	2.1	2.3

5*	F	42	2	24	Y	1.6	1.8	2.1	1.9

6*	F	34	3	24	Y	2.4	2.7	2.1	2.0

7	F	49	4	48	Y	3.1	2.9	2.7	2.2

8*	F	49	1	48	Y	0.03	1.5	1.2	1.3

9	F	50	2	24	Y	2.2	2.1	2.4	2.5

10*	F	37	4	48	Y	6.5	4.5	5.2	2.3

11	F	43	4	48	Y	1.8	1.9	2.3	2.1

12	F	42	3	24	Y	2.1	2.3	1.7	2.5

13*	F	43	1	48	N	10.5	2.3	1.8	2.4

14*	F	51	3	24	Y	8.8	4.0	3.8	2.3

15	M	57	1	48	N	2.2	2.7	1.9	1.2

16	F	38	3	24	Y	1.9	2.2	2.3	3.7

17	M	57	1	48	Y	4.5	2.5	3.6	2.8

18	F	37	3	24	Y	3.4	3.3	2.5	2.8

*; Cases 5 and 6 required short-term thyroxine supplement, case 8 progressed to develop post-interferon Graves' like thyrotoxicosis, cases 10 and 14 had subclinical hypothyroidism and case 13 was in the hypothyroid phase at the end of treatment. SVR; Sustained virologic response, Rx; Treatment, TSH; Thyrotropin.

### Thyroid function assessments

All patients were assessed by an endocrinology team and confirmed to have thyroiditis whilst receiving interferon therapy as previously reported [7]. At the end of interferon treatment, all had baseline thyroid function studies and thyroid autoantibodies, at 12, 24 and 36 months post therapy. Follow-ups at 24 and 36 months were performed by mail correspondence and telephone interviews. Patients were recalled and reviewed formally if clinically indicated.

### Laboratory assay characteristics

Third generation serum TSH, serum fT4 and fT3 were determined by two-site sandwich immunoassay using an automated chemiluminescent system (Diagnostic Products Corporation, Immulite 2000). The reference range (RR) for TSH was 0.4-4.0 mU/L, fT4 10.0-26.0 and fT3 3.5-5.5 pmol/L. The coefficients of variation (CV) were 5.0% and 5.1% at TSH concentrations of 4.0 mU/L and 10.0 mU/L respectively. For fT4, the CV was 6.5% at 10.0 pmol/L and fT3 8.9% at 3.5 pmol/L.

Serum autoantibodies to thyroglobulin and thyroperoxidase were measured by agglutination (Serodia-ATG and Serodia-AMC, Fujirebio, Inc., Tokyo, Japan). Titres of less than 1:40 were considered normal for both.

Thyroid Stimulating Immunoglobulin was measured using cell culture and radio-immunoassay. This is an in-house bioassay using Chinese Hamster Ovary (CHO) cells in culture to detect the presence of thyroid stimulating activity. The CHO cells are transfected with the TSH receptor genes and thus are responsive to TSI. Thyroid-stimulating activity is measured by evaluating the intracellular release of cAMP induced by the patient's serum immunoglobulin on the CHO cells. The results are reported as units/mL (U/mL). TSI should be absent in the normal population. A TSI level of <10 is considered negative, 10-50 as weakly, 50-100 as moderately and >100 U/mL as strongly positive.

### Duration of follow-ups

Duration of follow-ups started from the completion of interferon treatment, be it 24 or 48 weeks.

### Definition of thyroiditis

Thyroiditis is defined as the triad of clinical and/or biochemical thyrotoxicosis followed by a hypothyroid phase, with an initial reduced/negligible thyroid pertechnetate uptake scan. All uptake scans were reviewed by a specialist nuclear physician consultant. Thyroid autoantibodies may be present but are not considered essential to the diagnosis.

## Results

There were 18 cases of thyroiditis available for the study. All recovered from their TD at the end of the study. Cases 5 and 6 required short-term thyroxine supplement but subsequently were successfully withdrawn from thyroxine. Case 8 progressed to developed temporary Graves-like thyrotoxicosis demonstrating the uncommon tri-phasic thyroiditis that had previously been reported [8,9]. In addition, cases 10 and 14 had subclinical hypothyroidism which resolved with time and did not require thyroxine at any stage, Table 2. Similarly, case 13 was in the hypothyroid phase of the thyroiditis at the end of treatment, was treated expectantly and resolved without thyroxine. At 36 months, all cases had normal thyrotropin levels and all thyroid autoantibodies were undetectable. No patient required thyroxine at the end of the study.

**Table 2:** Auto-antibody profiles in 18 thyroiditis patients.

***Subject*** ***No*.**	***End of Rx anti-TPO***	***Anti-TPO at 12 months***	***Anti-TPO at 24 months***	***Anti-TPO at 36 months***	***End of Rx anti-Tg***	***Anti-Tg at 12 months***	***Anti-Tg at 24 months***	***Anti-Tg at 36 months***	***End of Rx TSI***	***TSI at 12 months***	***TSI at 24 months***	***TSI at 36 months***
		
1	<1	<1	<1	<1	<1	<1	<1	<1	<10	<10	<10	<10
		
2	<1	<1	<1	<1	<1	<1	<1	<1	<10	<10	<10	<10
		
3	<1	<1	<1	<1	<1	<1	<1	<1	<10	<10	<10	<10
		
4	<1	<1	<1	<1	<1	<1	<1	<1	<10	<10	<10	<10
		
5*	1024	<1	<1	<1	<1	<1	<1	<1	<10	<10	<10	<10
		
6*	256	<1	<1	<1	<1	<1	<1	<1	<10	<10	<10	<10
		
7	<1	<1	<1	<1	<1	<1	<1	<1	<10	<10	<10	<10
		
8*	16	<1	<1	<1	<1	<1	<1	<1	19	10	<10	<10
		
9	<1	<1	<1	<1	<1	<1	<1	<1	<10	<10	<10	<10
		
10*	<1	<1	<1	<1	<1	<1	<1	<1	<10	<10	<10	<10
		
11	<1	<1	<1	<1	<1	<1	<1	<1	<10	<10	<10	<10
		
12	<1	<1	<1	<1	<1	<1	<1	<1	<10	<10	<10	<10
		
13*	<1	<1	<1	<1	<1	<1	<1	<1	<10	<10	<10	<10
		
14*	16	<1	<1	<1	<1	<1	<1	<1	<10	<10	<10	<10
		
15	<1	<1	<1	<1	<1	<1	<1	<1	<10	<10	<10	<10
		
16	<1	<1	<1	<1	<1	<1	<1	<1	<10	<10	<10	<10
		
17	<1	<1	<1	<1	<1	<1	<1	<1	<10	<10	<10	<10
		
18	<1	<1	<1	<1	<1	<1	<1	<1	<10	<10	<10	<10

*; see Table 1 legend. Anti-TPO; anti-thyroperoxidase antibody, Anti-Tgl; anti-thyroglobulin antibody, TSI; Thyrotropin stimulating immunoglobulins.

With the exception of case 8, thyroid autoantibody titres declined towards normal at the end of treatment and remained unaltered throughout the duration of follow-up.

## Discussion

This is the first report to prospectively investigate the natural history of patients who develop thyroiditis during treatment with pegylated interferon-α and ribavirin for chronic hepatitis C infection. It is reassuring to ascertain that this form of TD is benign and that there is no long term recurrence or hypothyroidism. This benign prognosis is encouraging for both patients and clinicians. There appears to be little need to monitor for associated thyroid condition beyond 36 months after completion of treatment.

This benign outcome also parallels the thyroid autoantibodies which similarly resolved. With the exceptions of one patient (case 8), all thyroid antibodies normalise. In this affected case, autoantibodies persisted at the end of treatment suggesting ongoing immunomodulation but the consequent mild Graves' disease resolved after 12 months.

The involution of thyroid disease after interferon exposure is as expected and is probably due to two reasons. Firstly, it is because of the pharmacokinetics of the pegylated IFN which is expected to clear within 4 to 6 weeks. As a result, most of the TDs that occur post-therapy tend to do so in this time frame [10]. Secondly, once beyond the 6 month period where sustained virologic response (SVR) had been attained, the immunomodulating effect of the hepatitis C viral particle has been removed and hence its influence on thyroid tissues is no longer pertinent [11].

However our findings are inconsistent with other retrospective reports. Carella et al [12] studied a group of 114 chronic hepatitis C patients after an average of 6.2 years of follow-up and found some persistence of thyroid autoantibodies after 6.2 years of follow-up but without clinical findings of TDs. However, these patients were treated with IFN-α alone for 12 months. However the treatment was monotherapy with regular IFN. Vezali et al [13] described 2 cases which developed thyroiditis within 1 month of treatment completion and another 3 cases at 6, 6.5 and 26 months respectively. No further details were forthcoming. The frequency of thyroid testings was not mentioned during this post-treatment period. Jamil et al [6] looked at 45 cases of thyroid disease over a 12 year period but did not comment on the long-term outcomes of thyroid diseased patients nor the frequency of thyroid monitoring. Generally speaking in other retrospective series, the thyroid follow-ups are often ad hoc as a result and the outcome is not uniform and inconsistent [14,6]. The thyroid surveillance frequency is also inadequate to assess for the thyroid condition.

Most series report hypothyroidism as the commonest thyroid disorder in this setting but our experience has been that of thyroiditis almost exclusively [7]. In this setting, the prevalence of permanent hypothyroidism has been reported at ~50% [13] but where attempts or trials of thyroxine withdrawal were done, none of our patients required long term supplements. This suggests that the thyroid has completely recovered from the interferon-elicited injury and that hypothyroidism occurring in this setting deserved a trial of withdrawal before being committed to life-long therapy and all its potential complications.

The presence of high thyroid autoantibody titres also heavily influences the involution of thyroid diseases. Where they persisted or evolved, clinical diseases appear to follow as evident in cases 5, 6 and 8. However, because of the sporadic nature of post-treatment thyroid diseases, it remains uncertain whether routine thyroid autoantibodies should be performed routinely in this setting.

The major limitation of this study is the small number of subjects due the low prevalence of the condition. The second concern is the testing frequency of 12 month intervals during which it is conceivable that thyroid function may have fluctuated during this time. Regardless, it appears that thyroid disease sustained during interferon therapy for chronic hepatitis C infection is self-limiting and without any long last consequences. All cases recovered, compared with de novo thyroiditis where the progression to hypothyroidism can be as high as 20% in both adults and children [15,16]. Although there is no data beyond 3 years, it can safely be assumed that any development of thyroid conditions beyond this time should be deemed unrelated and treated each on its own merit. Perhaps monitoring for thyroid disease could be safely ceased at 6 months follow-up, coinciding with the SVR review.

## Conclusion

Despite the small number, the long term outcome of interferon induced thyroiditis appears benign. There is no long term risk of hypothyroidism. Larger studies will be needed to confirm this outcome. If this is the case, perhaps there is little need to follow these patients longer than 6 months after the completion of interferon therapy. This is to cover for any immediate thyroid conditions that may occur during this time frame. Follow-ups beyond 3 years appear unwarranted although this time frame is completely arbitrary.

## Competing interests

The authors declare that they have no competing interests.

## Authors' contributions

HAT conceived the study, participated in its design, assisted with data collection and statistical analysis, and coordinated and helped to draft the manuscript. GEMR contributed to the statistical and meta-analytical methods, and participated in the discussion and drafting of the manuscript. TLJ, EAI gathered, provided the data, and participated in the discussion and drafting of the manuscript. All authors read and approved the final revised manuscript.
